# Assessment of Low-Density Lipoprotein Cholesterol (LDL-C) Target Attainment in High-Risk Patients Receiving Statin Plus Ezetimibe Therapy: A Retrospective Cross-Sectional Study

**DOI:** 10.7759/cureus.87963

**Published:** 2025-07-15

**Authors:** Syed Salah Ud Din Umer Khatab Gillani, Azaz Ahmad Khan, Mariam Mobusher, Mazhar Khalil, Saeed Ullah, Arif Ullah, Bibi Khalida Hussain, Malik Muaz Iqbal, Dure Nayab, Naqeeb Ullah

**Affiliations:** 1 Department of Internal Medicine, District Headquarters (DHQ) Teaching Hospital, Dera Ismail Khan, PAK; 2 Department of General Medicine, Life Medical Care Hospital, Swabi, PAK; 3 Department of General Surgery, Shalamar Hospital, Lahore, PAK; 4 Department of Medicine, Hayatabad Medical Complex Peshawar, Peshawar, PAK; 5 Department of Internal Medicine, Saidu Group of Teaching Hospitals, Swat, PAK; 6 Department of Internal Medicine, Lady Reading Hospital, Peshawar, PAK; 7 Department of Internal Medicine, Mayo Hospital, Lahore, PAK

**Keywords:** cardiovascular risk, ezetimibe, “high-risk” individuals, “ldl-c”, lipid management, statin medication, target achievement

## Abstract

Introduction

Achieving optimal low-density lipoprotein cholesterol (LDL-C) reduction in high-risk patients remains a challenge, even with combination lipid-lowering therapy. This study evaluated LDL-C target attainment (≤70 mg/dL per European Society of Cardiology/European Atherosclerosis Society (ESC/EAS) 2019 guidelines) in high-risk patients receiving statin plus ezetimibe therapy at a single tertiary care center in Peshawar, Pakistan.

Methodology

A retrospective analysis of a cross-sectional dataset was conducted at Hayatabad Medical Complex (HMC) over 12 months. A total of 123 high-risk patients, as defined by ESC/EAS 2019 criteria, who had been on statin plus ezetimibe therapy for a minimum of three months (mean duration: 4.2 ± 1.1 months), were included. LDL-C levels were recorded at a single post-treatment follow-up time point. Paired t-tests were used to assess LDL-C changes, and chi-square tests along with multivariable logistic regression were employed to identify predictors of target attainment.

Results

Mean LDL-C decreased significantly from 156.3 ± 32.5 mg/dL to 84.7 ± 24.1 mg/dL (mean reduction: 71.6 mg/dL; 95% CI: 66.8-76.4; p < 0.001). Overall, 53 of 123 patients (43.1%; 95% CI: 34.6-51.8%) achieved the LDL-C target. Target attainment was lower among diabetic patients (24 of 71; 33.8%) and smokers (13 of 39; 33.3%). In multivariable analysis, diabetes (aOR: 0.52; 95% CI: 0.28-0.97) and smoking (aOR: 0.55; 95% CI: 0.27-0.98) were independently associated with lower target attainment. High-intensity statin use was positively associated with achieving LDL-C goals (33 of 68; 48.5%; aOR: 1.89; 95% CI: 1.02-3.49).

Conclusion

This single-center study highlights that despite combination therapy, more than half of high-risk patients failed to achieve LDL-C targets. The findings underscore the need for more individualized strategies, improved adherence, and possibly adjunctive therapies. Although the data are from a single center in Peshawar, the trends reflect common challenges in real-world lipid management across similar low- to middle-income healthcare settings.

## Introduction

Cardiovascular diseases (CVDs) remain the leading cause of morbidity and mortality worldwide, accounting for an estimated 17.9 million deaths annually [1]. Atherosclerotic cardiovascular disease (ASCVD), driven primarily by elevated low-density lipoprotein cholesterol (LDL-C), is a key contributor to this burden, with LDL-C accumulation accelerating plaque formation and increasing the risk of coronary artery disease (CAD), stroke, and other vascular events [2,3].

Statins, or 3-Hydroxy-3-Methylglutaryl-Coenzyme A (HMG-CoA) reductase inhibitors, form the cornerstone of LDL-C-lowering therapy and have significantly reduced cardiovascular events across diverse populations [4]. However, many high-risk patients, such as those with diabetes, chronic kidney disease (CKD), or established CAD, do not achieve recommended LDL-C targets with statin monotherapy alone [5,6]. For such patients, ezetimibe, a non-statin agent that inhibits intestinal cholesterol absorption, offers an additional 20-25% LDL-C reduction when combined with statins [7]. Ezetimibe is widely used in Pakistan and other South Asian countries due to its affordability, favorable side effect profile, and availability on standard hospital formularies [8].

This study adopts the European Society of Cardiology/European Atherosclerosis Society (ESC/EAS) 2019 guidelines for lipid management, which recommend LDL-C targets of ≤70 mg/dL for high-risk patients and ≤55 mg/dL for very high-risk individuals [9]. These guidelines are widely referenced in South Asian clinical practice owing to their evidence-based risk stratification and adaptability across various healthcare systems. High-risk and very high-risk classifications in this study follow ESC/EAS 2019 definitions, including patients with ASCVD, diabetes with organ damage, CKD, or a SCORE ≥10% [10].

While combination lipid-lowering therapy is increasingly prescribed in Pakistan, there is limited real-world data on LDL-C target attainment in this setting. Most available studies originate from Western countries, and local differences in treatment practices, adherence patterns, and comorbidity profiles may affect generalizability [11]. This study addresses this knowledge gap by evaluating LDL-C goal achievement among high-risk patients on statin plus ezetimibe therapy at Hayatabad Medical Complex (HMC), a large tertiary care hospital in Peshawar, Pakistan.

The primary aim is to determine the proportion of high-risk patients who achieve LDL-C targets per ESC/EAS 2019 criteria after at least three months of combination therapy. We also assess clinical and demographic predictors of LDL-C target attainment. These findings will help inform tailored lipid-lowering strategies in South Asia and contribute to improved ASCVD risk management.

## Materials and methods

This was a single-center, retrospective cross-sectional study conducted at the Department of Cardiology, Hayatabad Medical Complex (HMC), Peshawar, a major regional referral center with an annual outpatient cardiology volume exceeding 40,000 patients. The study was conducted over a 12-month period from January to December 2023. In this hybrid design, retrospective data were used to analyze LDL-C levels at a single follow-up point after at least three months of statin plus ezetimibe therapy, consistent with a cross-sectional outcome assessment framework.

Sample size and sampling technique

A sample size of 123 patients was calculated using the WHO sample size calculator with a 95% confidence level, 5% margin of error, and an expected LDL-C target attainment rate of 50%, used as a conservative estimate given regional variability in reported outcomes. The equation used was $\text{n} = \frac{Z^{2}.p (1-p)}{d^{2}}$, where Z = 1.96, p = 0.50, and d = 0.05. Patients were selected using a non-probability consecutive sampling technique. To reduce selection bias, all eligible cases during the study period were included without restriction.

Eligibility criteria

Patients aged ≥18 years, classified as high or very high risk for ASCVD according to the ESC/EAS 2019 guidelines, were eligible. This included those with established ASCVD, diabetes with target organ damage, CKD, or a calculated Systematic COronary Risk Evaluation (SCORE) ≥10%. Inclusion required confirmed use of a statin plus ezetimibe regimen for a minimum of three months and availability of follow-up LDL-C levels. Patients on other lipid-lowering therapies, with incomplete or missing records, or with secondary causes of dyslipidemia (e.g., hypothyroidism, nephrotic syndrome) were excluded. Medication compliance was verified using electronic prescription records and pharmacy refill logs.

Definitions and data handling

Statin intensity was categorized as per ESC/EAS 2019 guidelines: high-intensity statins included atorvastatin ≥40 mg/day or rosuvastatin ≥20 mg/day, while lower doses were classified as moderate-intensity. The mean treatment duration before LDL-C reassessment was 4.2 ± 1.1 months. Patients were stratified by risk category, and LDL-C targets were applied accordingly: <70 mg/dL for high-risk and <55 mg/dL for very high-risk patients. Both thresholds were analyzed in subgroup assessments. Missing data were handled using complete-case analysis; no imputation was performed. Only records with complete variables of interest were included.

Data analysis

Data were analyzed using SPSS version 26. Continuous variables were reported as means ± SDs, and categorical variables as frequencies and percentages. Paired t-tests were used to compare LDL-C levels before and after treatment. Chi-square tests were applied to compare LDL-C goal attainment across clinical subgroups. Multivariable logistic regression was performed to identify independent predictors of LDL-C target attainment. A p-value <0.05 was considered statistically significant.

Ethical considerations

Ethical approval for this study was secured from the Institutional Review Board (IRB) of the institution. Patient confidentiality and privacy were rigorously upheld by anonymizing data obtained from medical records. Informed permission was not required due to the retrospective design of the study, which employed pre-existing data. All information was managed in accordance with applicable ethical standards, including those outlined in the Declaration of Helsinki, to safeguard patients’ rights and welfare.

## Results

A total of 123 patients satisfied the inclusion criteria and were incorporated into the final analysis. The average age of the patients was 58.7 ± 11.2 years, ranging from 32 to 82 years. Among the participants, 76 (61.8%) were male, and 47 (38.2%) were female. Regarding comorbidities, 68 (55.3%) were diagnosed with diabetes mellitus, 82 (66.7%) with hypertension, 39 (31.7%) had a history of smoking, 26 (21.1%) had chronic renal disease, and 71 (57.7%) had a history of CAD. A considerable percentage of individuals possessed multiple risk factors known to contribute to the progression of CVD. The baseline demographic and clinical parameters are summarized in Table 1.

**Table 1:** Baseline demographic and clinical characteristics of patients (n = 123). CAD: Coronary artery disease.

Variable	Frequency (n)	Percentage (%)
Age (mean ± SD)	58.7 ± 11.2	N/A
Gender		
Male	76	61.80%
Female	47	38.20%
Diabetes mellitus	68	55.30%
Hypertension	82	66.70%
Smoking history	39	31.70%
Chronic kidney disease	26	21.10%
History of CAD	71	57.70%

The mean baseline LDL-C level before starting statin plus ezetimibe therapy was 156.3 ± 32.5 mg/dL, which decreased to 84.7 ± 24.1 mg/dL after at least three months of combination therapy. This reflected a mean LDL-C reduction of 71.6 ± 18.2 mg/dL, which was statistically significant (p < 0.001, paired t-test). According to the ESC/EAS 2019 guidelines, 53 patients (43.1%) achieved their LDL-C target levels, while 70 (56.9%) did not meet the recommended goal. These results underscore the challenges in achieving optimal LDL-C control, despite substantial reductions with statin plus ezetimibe therapy. The findings are summarized in Table 2.

**Table 2:** LDL-C target attainment and LDL-C levels before and after therapy (n = 123). LDL-C: Low-density lipoprotein cholesterol.

Variable	Value
Baseline LDL-C (mean ± SD)	156.3 ± 32.5 mg/dL
Follow-up LDL-C (mean ± SD)	84.7 ± 24.1 mg/dL
Mean reduction in LDL-C (mean ± SD)	71.6 ± 18.2 mg/dL
p-value for LDL-C reduction	< 0.001
Achieved LDL-C target (n, %)	53 (43.1%)
Did not achieve LDL-C target (n, %)	70 (56.9%)

A subgroup analysis was conducted to examine factors associated with LDL-C target attainment (Table 3). Patients without diabetes mellitus were significantly more likely to achieve the LDL-C target than those with diabetes (55.8% vs. 33.8%; χ² = 4.35, df = 1, p = 0.037). Similarly, non-smokers demonstrated a higher target attainment rate than smokers (47.6% vs. 33.3%; χ² = 4.17, df = 1, p = 0.041). No significant association was observed between gender and LDL-C control, as 43.4% of males and 42.6% of females achieved the target (χ² = 0.01, df = 1, p = 0.912). Hypertension status also did not significantly impact target attainment (46.3% in non-hypertensive vs. 41.5% in hypertensive patients; χ² = 0.26, df = 1, p = 0.611). Additionally, there was no significant difference in LDL-C goal achievement between patients with and without chronic kidney disease (34.6% vs. 45.4%; χ² = 1.12, df = 1, p = 0.289).

**Table 3:** Association of clinical characteristics with LDL-C target attainment. Percentages are based on subgroup totals (e.g., the percentage of diabetic patients who achieved the LDL-C target out of all diabetic patients). p < 0.05 was considered statistically significant. Chi-square (χ²) tests with 1 degree of freedom were used for all comparisons. LDL-C: Low-density lipoprotein cholesterol.

Variable	Achieved n (% of subgroup)	Not achieved n (% of subgroup)	χ²	p-value
Gender				
Male (n = 76)	33 (43.4%)	43 (56.6%)	0.01	0.912
Female (n = 47)	20 (42.6%)	27 (57.4%)		
Diabetes mellitus				
Yes (n = 71)	24 (33.8%)	47 (66.2%)	4.35	0.037*
No (n = 52)	29 (55.8%)	23 (44.2%)		
Hypertension				
Yes (n = 82)	34 (41.5%)	48 (58.5%)	0.26	0.611
No (n = 41)	19 (46.3%)	22 (53.7%)		
Smoking history				
Smoker (n = 39)	13 (33.3%)	26 (66.7%)	4.17	0.041*
Non-smoker (n = 84)	40 (47.6%)	44 (52.4%)		
Chronic kidney disease				
Yes (n = 26)	9 (34.6%)	17 (65.4%)	1.12	0.289
No (n = 97)	44 (45.4%)	53 (54.6%)		

**Figure 1:**
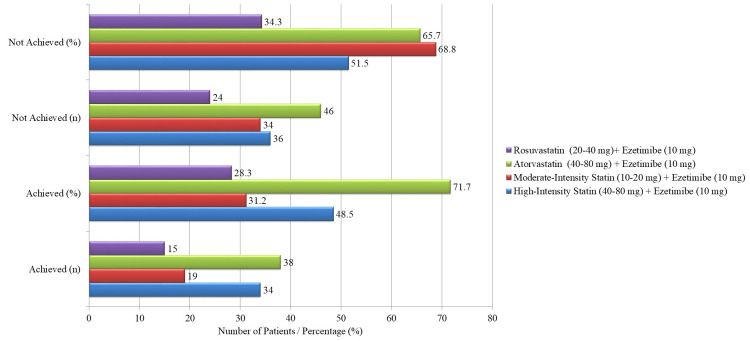
LDL-C target attainment based on statin type and intensity. LDL-C: Low-density lipoprotein cholesterol.

When evaluating the type and intensity of statin therapy, it was observed that high-intensity statin use (e.g., atorvastatin 40-80 mg or rosuvastatin 20-40 mg) in combination with ezetimibe resulted in higher LDL-C target attainment (48.5%) compared to moderate-intensity statin use (31.2%, p = 0.029). Patients on high-intensity statin therapy were more likely to achieve LDL-C goals than those on moderate-intensity therapy. Furthermore, while patients on atorvastatin plus ezetimibe achieved LDL-C goals more frequently than those on rosuvastatin plus ezetimibe, the difference was not statistically significant (71.7% vs. 28.3%, p = 0.158). These findings suggest that statin intensity plays a crucial role in LDL-C target attainment. The relationship between statin type/intensity and LDL-C attainment is illustrated in Figure 1.

## Discussion

In this study, we assessed LDL-C target attainment among high-risk patients receiving statin plus ezetimibe therapy at Hayatabad Medical Complex (HMC), a major tertiary care referral center in Peshawar, Pakistan. Notably, this represents one of the first hospital-based investigations from the region evaluating LDL-C control using combination lipid-lowering therapy in a South Asian population. The results showed that only 43.1% of patients achieved the LDL-C target recommended by the 2019 ESC/EAS guidelines (i.e., ≤70 mg/dL for high-risk and ≤55 mg/dL for very high-risk patients), despite at least three months of combination therapy. A significant mean LDL-C reduction of 71.6 ± 18.2 mg/dL was observed; yet, more than half of the patients (56.9%) failed to achieve the recommended targets. These findings highlight the challenges of implementing guideline-based lipid targets in real-world settings, especially within resource-constrained healthcare systems.

When contextualized with previous studies, our target attainment rate of 43.1% falls within the reported global range of 30%-50% among high-risk patients treated with statin-ezetimibe combinations. Similar patterns of suboptimal LDL-C control despite dual therapy have been consistently reported in observational and registry-based studies across diverse populations [12,13]. The observed LDL-C reduction is also consistent with prior reports, where combination therapy typically yields reductions of 65-75 mg/dL [14].

Similarly, patients receiving high-intensity statin therapy were more likely to achieve LDL-C goals than those on moderate-intensity regimens, consistent with recommendations for very high-risk patients [15]. Although our comparison of atorvastatin- and rosuvastatin-based combinations did not reach statistical significance, previous data suggest a modest LDL-C benefit with atorvastatin, aligning with 2019 ESC/EAS recommendations for aggressive lipid targets and adjunctive therapies in very high-risk patients with uncontrolled lipids [16]. This may reflect underlying pharmacogenetic and metabolic variability, which could be particularly relevant in South Asian populations [17].

When contextualized with previous studies, our target attainment rate of 43.1% falls within the reported global range of 30%-50% among high-risk patients treated with statin-ezetimibe combinations [12,13]. The observed LDL-C reduction is also consistent with prior reports, where combination therapy typically yields reductions of 65-75 mg/dL [14]. However, baseline LDL-C plays a crucial role in determining the likelihood of achieving absolute targets, patients with higher starting LDL-C levels may require intensified or prolonged therapy, underscoring the need for individualized treatment approaches.

Importantly, the study lacked objective adherence data (e.g., pharmacy refill records or pill counts), which limits the ability to differentiate between true pharmacologic non-response and medication non-adherence. Moreover, treatment intensification may have been delayed in some patients, reflecting therapeutic inertia, an underrecognized barrier to achieving lipid goals in routine practice. Access to additional lipid-lowering therapies such as PCSK9 inhibitors is restricted in the local setting due to high costs and limited formulary availability, despite their proven efficacy in high-risk populations [18].

Collectively, these findings highlight persistent gaps in lipid management and the need for strengthened systems to support risk-based therapy, timely treatment escalation, and patient adherence. Addressing these gaps through physician training, patient education, and healthcare system reform may significantly improve LDL-C target attainment and reduce cardiovascular risk in high-risk South Asian populations.

Limitations and future suggestions

This study has several limitations. First, the retrospective cross-sectional design limits causal inference and precludes the assessment of temporal changes in lipid levels. Although multivariable analysis was performed, unmeasured confounders, such as dietary habits, physical activity, and socioeconomic status, may have influenced LDL-C outcomes. Furthermore, the study lacked objective adherence data (e.g., pharmacy refill records), making it difficult to distinguish between pharmacologic non-response and medication non-adherence. The absence of data on the duration and severity of comorbidities like diabetes and hypertension also limits the ability to explore their full impact on lipid metabolism.

Although we recorded baseline LDL-C values, a stratified analysis to assess how these influenced the likelihood of achieving LDL-C targets was not conducted. Additionally, other lipid parameters, such as non-HDL cholesterol, triglycerides, and apolipoprotein B, were not analyzed, which could have provided a more comprehensive assessment of cardiovascular risk. The study’s single-center design and modest sample size may also limit generalizability, and the use of non-probability consecutive sampling could introduce selection bias, despite our efforts to include all eligible records over a defined period.

Future research should prioritize prospective, multicenter studies with larger and more diverse populations. Integration of structured real-world data collection, such as electronic adherence monitoring, risk stratification tools, and longer-term follow-up, will be critical. There is also a need to evaluate the cost-effectiveness and implementation feasibility of newer lipid-lowering agents, including PCSK9 inhibitors, in high-risk South Asian populations. Lastly, public health interventions aimed at improving treatment adherence, physician awareness, and patient education may substantially enhance LDL-C control in clinical practice.

## Conclusions

This study demonstrates that while combination therapy with statins and ezetimibe significantly lowers LDL-C levels in high-risk patients, a substantial proportion still fail to achieve guideline-recommended targets. Clinical characteristics such as diabetes mellitus and smoking were associated with lower LDL-C target attainment, indicating the need for stratified management approaches in patients with complex comorbidities. In contrast, higher success rates among patients on high-intensity statin therapy underscore the importance of initiating appropriately intensive regimens in very high-risk groups.

The findings highlight the ongoing challenge of translating lipid-lowering efficacy into real-world goal attainment, particularly in resource-limited settings. These results can inform local clinical guidelines by supporting early intensification of therapy and targeted patient education strategies to improve adherence and treatment response. In cases where statin-ezetimibe therapy proves insufficient, the role of newer lipid-lowering agents such as PCSK9 inhibitors or bempedoic acid may be explored, particularly in patients with persistently elevated LDL-C despite optimized standard therapy. Given the cross-sectional and retrospective nature of this study, the observed associations cannot confirm causality. Prospective, multicenter studies and interventional trials are warranted to validate these findings, explore underlying mechanisms, and guide the development of cost-effective, evidence-based lipid management pathways tailored to high-risk populations in South Asia and similar settings.
